# Nitrous Oxide

**Published:** 1882-10

**Authors:** 


NITROUS OXIDE.—That this is the safest of all anæsthetics has been established beyond a question. In one institution where such administration is subject of record, this gas has been given over 100,000 times, and not only without a death, but without causing in a single instance symptoms sufficiently serious to necessitate transporting the patient home in a carriage.

